# Multiplexed Quantification of First-Trimester Serum Biomarkers in Healthy Pregnancy

**DOI:** 10.3390/ijms26167970

**Published:** 2025-08-18

**Authors:** Natalia Starodubtseva, Alisa Tokareva, Alexey Kononikhin, Alexander Brzhozovskiy, Anna Bugrova, Evgenii Kukaev, Alina Poluektova, Vladimir Frankevich, Evgeny Nikolaev, Gennady Sukhikh

**Affiliations:** 1 V.I. Kulakov National Medical Research Center for Obstetrics Gynecology and Perinatology, Ministry of Healthcare of Russian Federation, 117997 Moscow, Russia; alisa.tokareva@phystech.edu (A.T.); as.kononikhin@gmail.com (A.K.); agb.imbp@gmail.com (A.B.); a_bugrova@oparina4.ru (A.B.); e_kukaev@oparina4.ru (E.K.); a_poluektova@oparina4.ru (A.P.); v_frankevich@oparina4.ru (V.F.); g_sukhikh@oparina4.ru (G.S.); 2 Project Center of Omics Technologies and Advanced Mass Spectrometry, 121205 Moscow, Russia; 3 Emanuel Institute of Biochemical Physics, Russian Academy of Sciences, 119334 Moscow, Russia; 4 V.L. Talrose Institute for Energy Problems of Chemical Physics, N.N. Semenov Federal Research Center for Chemical Physics, Russian Academy of Sciences, 119334 Moscow, Russia; 5 Moscow Center for Advanced Studies, 123592 Moscow, Russia; 6 Laboratory of Translational Medicine, Siberian State Medical University, 634050 Tomsk, Russia; 7 Department of Obstetrics, Gynecology, Perinatology and Reproductology, Institute of Professional Education, Federal State Autonomous Educational Institution of Higher Education I.M. Sechenov First Moscow State Medical University of the Ministry of Health of the Russian Federation, 119991 Moscow, Russia

**Keywords:** proteomics, blood, serum, pregnancy, healthy volunteers, biomarkers, MRM, mass spectrometry

## Abstract

The maternal circulating proteome reflects critical physiological adaptations during pregnancy, yet standardized reference profiles for early gestation are lacking. In this prospective study, we employed targeted liquid chromatography–multiple reaction monitoring–mass spectrometry (LC-MRM-MS) with stable isotope-labeled (SIS) standards to characterize the serum proteome of 83 women with uncomplicated singleton pregnancies between 11^+2^ and 13^+6^ weeks’ gestation. Robust analysis quantified 115 proteins (83% of targets), with 101 meeting ICH M10 standards. These included 38 FDA-approved, 19 CVD-related, and 25 CLIA-approved biomarkers. We identified 43 proteins significantly associated (*p* < 0.05) with gestational age, maternal factors (BMI, age, parity, and myomas), and fetal sex. Key findings included identification of 12 proteins significantly associated with trisomy risk (|R| = 0.21–0.45, *p* < 0.05) and extreme physiological variability in pregnancy zone protein (PZP, 123.9-fold), followed by apolipoprotein (a) (LPA; 9.9-fold) and pregnancy-associated plasma protein A (PAPP-A, 9.3-fold). In contrast, hemopexin (HPX) demonstrated remarkable stability (CV = 8.5%), suggesting its utility as a reference marker. The study successfully implemented multiples of the median (MoM) transformation for clinical standardization of protein profiles, with RobNorm proving particularly effective for batch-effect correction in our dataset. These methodological advances, combined with the establishment of comprehensive pregnancy-specific reference ranges, provide a valuable foundation for future research. The optimized analytical framework and protein signatures identified in this work not only enable the development of next-generation screening approaches but also offer new insights into the molecular adaptations occurring during early pregnancy.

## 1. Introduction

Blood plasma and serum represent invaluable resources for biomarker discovery, offering a rich proteomic landscape that reflects both physiological and pathological states [1]. The minimally invasive nature of blood collection and the diverse protein origins within these biofluids make them particularly attractive for clinical diagnostics and mechanistic studies [2]. While substantial progress has been made in cataloging plasma proteins through initiatives like GOBIOM and MarkerDB, significant gaps remain in our understanding of how intrinsic factors such as age, sex, and ethnicity influence proteome variability [3,4,5,6,7,8]. This knowledge gap becomes particularly critical in pregnancy, where profound physiological adaptations render standard reference values inadequate [9,10].

The clinical potential of plasma/serum proteomics is evidenced by its applications across diverse conditions, from cardiovascular diseases to neurodegenerative disorders and pregnancy complications [11,12,13,14,15,16,17,18,19]. However, successful biomarker translation requires rigorous validation of accuracy, specificity, and reproducibility across populations [1,20,21]. This challenge is magnified in pregnancy by dynamic physiological changes that vary significantly with gestational age, necessitating specialized reference standards [6,22,23]. Notably, major resources like the Human Disease Blood Atlas lack pregnancy-specific data, underscoring this unmet need [24,25].

Mass spectrometry (MS) has revolutionized protein quantification, particularly through targeted methods using stable isotope standards (SIS) and multiplexed multiple reaction monitoring (MRM) panels capable of precise, high-throughput protein measurement [26,27,28,29,30]. Yet, two critical barriers remain for pregnancy applications: establishing gestational age-specific reference ranges [5,6,31,32] and developing robust normalization approaches to account for biological and technical variability [3,9,10,22,23,32].

The primary aim of this study was to establish clinically adjusted multiples of the median (MoM) references for 139 first-trimester serum proteins in normal pregnancy (*n* = 83) using rigorously validated LC-MRM-MS with natural synthetic proteotypic (NAT) and SIS tryptic peptides. By including 40 preeclampsia markers and developing standardized normalization approaches, we provide both immediate clinical tools and a foundational framework for pregnancy proteomics research [33].

## 2. Results

### 2.1. Clinical and Demographic Characteristics

This prospective study included 83 women with physiological pregnancies undergoing first-trimester screening. Participants had a median age of 30.5 years (range 20.5–37.3) and a BMI of 21.2 kg/m^2^ (range 15.6–30.1). Blood collection occurred at a median of 12.4 weeks’ gestation (range 11^+2^–13^+6^). Key findings included uterine myoma in 8 women (10%), anemia in 27 (33%), and near-equal fetal sex distribution (49% male). Parity distribution was 48% primiparous and 39% secundiparous. Biomarker analysis revealed median pregnancy-associated plasma protein A (PAPP-A) of 3.03 mIU/mL (0.95 MoM), placental growth factor (PIGF) of 25.3 pg/mL (0.84 MoM), and free β-human chorionic gonadotropin (free β-hCG) of 52.61 ng/mL (0.99 MoM). Median uterine artery PI (UtA-PI) was 1.57 (0.99 MoM), and mean arterial pressure (MAP) was 83.33 mmHg (0.98 MoM). Pregnancy risks were preeclampsia (PE) 1:1357, intrauterine growth restriction (IUGR) 1:554, and preterm delivery 1:2115 (median values). All data are detailed in Table 1.

### 2.2. Analytical Performance and Data Normalization

**Figure 1:**
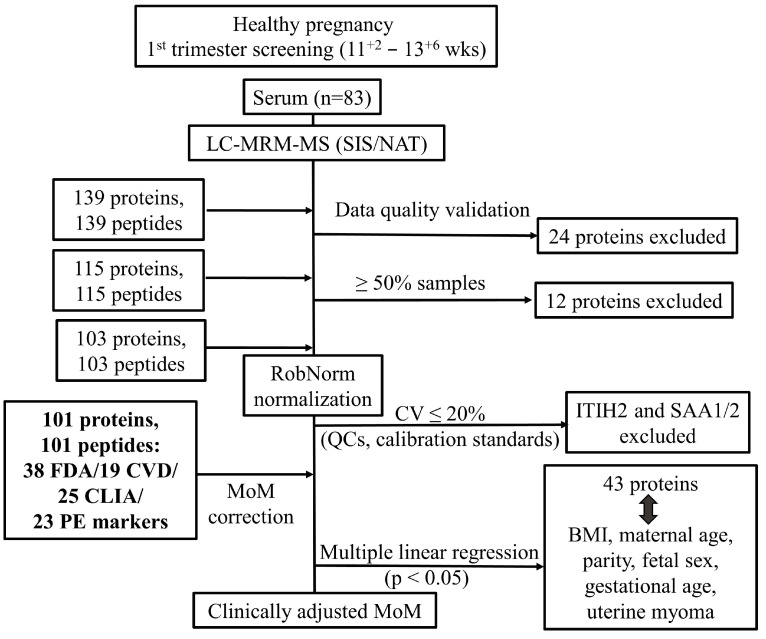
Development of reference ranges for first-trimester maternal serum proteins (11^+2^−13^+6^ weeks) using LC-MRM-MS with stable isotope-labeled standards (SIS) and native (NAT) peptides. Workflow includes normalization (quantile, LOESS, RobNorm, and ComBat), MoM correction, and adjustment for six maternal/fetal covariates. Clinically adjusted MoM reference ranges were established for 101 rigorously validated proteins. Pipeline of protein analysis, filtering, and transforming proteome data.

Among the 139 targeted proteins studied, 115 (82.7%) were reliably detected and quantified using LC-MRM-MS with corresponding SIS peptides (Supplementary Table S1). Twelve proteins—alpha-1-antitrypsin, apolipoprotein A-I, cholesteryl ester transfer protein, clusterin, complement C1q subcomponent subunit B, fibrinogen alpha chain, fibrinogen beta chain, fibrinogen gamma chain, kallistatin, keratin type I cytoskeletal 9, peroxiredoxin-2, and serum paraoxonase/lactonase 3—were excluded due to quantification in fewer than 50% of samples (Figure 1).

Median coefficient of variation (CV) in calibration standards (A–H) and quality controls (QCA-C and pooled sample serum, CLP) are summarized in Supplementary Table S2. For raw data, the median mean CV across proteins was 7.9%, with three proteins (inter-alpha-trypsin inhibitor heavy chain H2, ITIH2; inter-alpha-trypsin inhibitor heavy chain H1, ITIH1; and serum amyloid A-1 and A-2 proteins, SAA1/2) exhibiting mean CVs of 20% or higher (Figure S1A). Normalization methods yielded varying results. Quantile normalization increased the median mean CV to 24% (Figure S1B). In contrast, LOESS normalization maintained a low median mean CV of 7.7%, though four proteins (ITIH1, ITIH2, L-selectin, SELL, and SAA1/2) had CVs of at least 20% (Figure S1C). RobNorm normalization further reduced the median mean CV to 7.2%, with only two proteins (ITIH2 and SAA1/2) exceeding the 20% threshold (Supplementary Figure S1D). ComBat normalization resulted in the highest median mean CV at 30.3% (Supplementary Figure S1E).

Both LOESS and RobNorm normalization significantly reduced the mean CV compared to raw data (*p* < 0.001 for each; paired Mann–Whitney test). Additionally, these methods effectively removed batch-associated variation (Supplementary Figure S2). However, RobNorm outperformed LOESS normalization, yielding a lower mean CV (*p* < 0.001; paired Mann–Whitney test) and fewer proteins excluded due to excessive variation. These results suggest that RobNorm is the optimal method for batch-effect correction in this dataset.

### 2.3. First Trimester Serum Proteome Profiling

A total of 101 proteins met all study quality control criteria, demonstrating analytical performance compliant with ICH M10 guidelines for bioanalytical method validation [34]. These proteins included 38 FDA-approved biomarkers [35], 19 CVD markers [36,37], and 25 Clinical Laboratory Improvement Amendments (CLIA)-approved clinical targets [38]. Additionally, 23 proteins represented potential markers for PE, a severe pregnancy-associated disorder [33].

**Figure 2:**
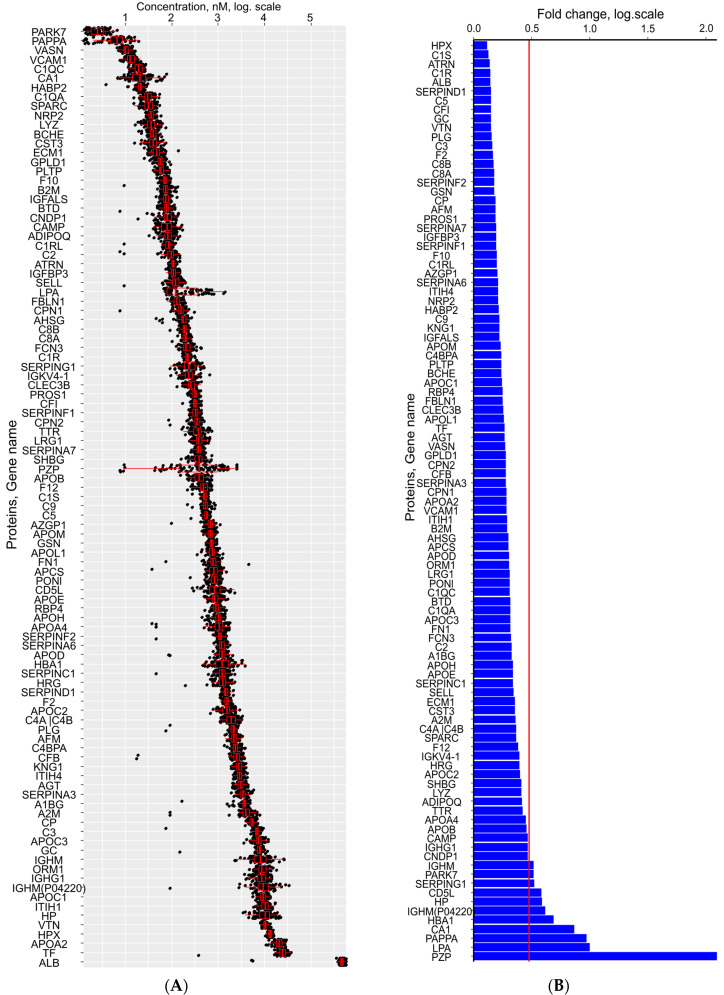
Protein concentration profiles after RobNorm normalization (*n* = 83). (**A**) Distribution metrics (range, central tendency). (**B**) Variability analysis (95th/5th percentile ratios; threshold = 3, red line).

Protein concentration analysis revealed a dynamic range spanning 5.3 orders of magnitude in both RobNorm-normalized and raw datasets (Figure 2A, Supplementary Figure S3A; Tables S3 and S4). In RobNorm-normalized data, the lowest concentrations were observed for protein deglycase DJ-1 (PARK7; 2.46 [1.81–3.43] nM), PAPP-A (6.58 [4.85–8.82] nM), and Vasorin (VASN; 10.08 [9.19–11.39] nM). Conversely, the highest concentrations were found for serum albumin (ALB; 4.73 × 10^5^ [4.36 × 10^5^–5.08 × 10^5^] nM), serotransferrin (TF; 2.45 × 10^4^ [2.18 × 10^4^–2.86 × 10^4^] nM), and apolipoprotein A-II (APOA2; 2.05 × 10^4^ [1.81 × 10^4^–2.37 × 10^4^] nM). The raw data showed comparable concentration ranges for these proteins, with PARK7 (2.32 [1.54–3.51] nM), PAPP-A (6.34 [4.39–8.47] nM), and VASN (9.88 [8.60–11.25] nM) at the lower end and ALB (4.54 × 10^5^ [4.29 × 10^5^–4.95 × 10^5^] nM), TF (2.37 × 10^4^ [2.03 × 10^4^–2.77 × 10^4^] nM), and APOA2 (2.00 × 10^4^ [1.78 × 10^4^–2.38 × 10^4^] nM) at the higher end.

Fold change analysis demonstrated that 90 proteins in RobNorm-normalized data showed minimal variation (fold change < 3). Pregnancy zone protein (PZP) exhibited the most dramatic change (123.9-fold), followed by apolipoprotein (a) (LPA; 9.9-fold), PAPP-A (9.3-fold), and carbonic anhydrase 1 (CA1; 7.3-fold). Hemopexin (HPX) was the most stable protein (1.3-fold change). Similar patterns were observed in raw data, where PZP showed a 155.8-fold change, followed by LPA (9.5-fold), PAPP-A (8.5-fold), and CA1 (6.6-fold), with 87 proteins demonstrating fold changes <3 and ALB being the most stable (1.4-fold change) (Figure 2B, Supplementary Figure S3B; Tables S4 and S5).

To align serum proteome analysis with clinical diagnostic standards, we applied MoM transformation. Following MoM transformation (Supplementary Figure S4), the central 90% of values (5th–95th percentiles) ranged from 0.65 MoM, with extreme values observed for HPX (0.28 MoM) and LPA (5.4 MoM) (Supplementary Table S6).

### 2.4. Associations Between Serum Proteome and Clinical Parameters

To assess first-trimester maternal serum proteome for potential clinical confounders, we performed Spearman’s rank correlation. This nonparametric approach is particularly appropriate for our analysis, as it accommodates both continuous variables (such as BMI and maternal age) and categorical variables (including fetal sex and parity). Our analysis revealed statistically significant associations (*p* < 0.05) between maternal serum protein levels and several key parameters, including maternal BMI, age, parity status, fetal sex, and sample collection timing. A particularly notable finding was the moderate inverse correlation between maternal age and A2M (R = −0.41, *p* < 0.001). A2M showed strong positive correlations with both adjusted risk levels and background risk for trisomy 18 (R = 0.41 and R = 0.44, respectively, *p* < 0.001 for both), as well as for trisomies 13 and 21 (R = 0.44 and R = 0.42, respectively, *p* < 0.001 for both) (Supplementary Figure S5).

Several other proteins exhibited weaker but statistically significant associations with trisomy risk. IGFBP3 showed consistent weak positive correlations with background risk for all three trisomies (R = 0.23, *p* = 0.04). SERPING1 demonstrated similar associations for trisomies 13 and 18 (R = 0.24, *p* = 0.03) and a slightly weaker correlation for trisomy 21 (R = 0.22, *p* = 0.02). Negative correlations were observed for CST3 (R = −0.22 to −0.23, *p* = 0.04–0.047), CFI (R = −0.24 to −0.25, *p* = 0.03), SERPIND1 (R = −0.27 to −0.28, *p* = 0.01), and CPN2 (R = −0.26, *p* = 0.01 across all trisomies).

Additional protein–trisomy associations were identified in the adjusted risk analysis. For trisomy 13, weak correlations were found with AZGP1 (R = 0.26, *p* = 0.02), A2M (R = 0.27, *p* = 0.02), and SERPINA3 (R = −0.26, *p* = 0.02). Trisomy 18 showed associations with SERPINF1 (R = 0.23, *p* = 0.04), APOA4 (R = 0.26, *p* = 0.02), SERPING1 (R = 0.28, *p* = 0.01), and AZGP1 (R = 0.24, *p* = 0.03) and negative correlations with CFI (R = −0.27, *p* = 0.01), SERPIND1 (R = −0.25, *p* = 0.02), C1QA (R = −0.22, *p* = 0.046), and CPN2 (R = −0.27, *p* = 0.01). Trisomy 21 demonstrated weak associations with PON1 (R = 0.23, *p* = 0.04), IGFBP3 (R = 0.22, *p* = 0.046), SERPING1 (R = 0.25, *p* = 0.02), and CFI (R = −0.26, *p* = 0.02).

Multiple linear regression analysis revealed significant associations between maternal characteristics and concentrations of 43 proteins (Table 2, Supplementary Table S7). BMI and parity showed the strongest effects, influencing 21 and 13 proteins, respectively. Fetal sex significantly modulated concentrations of 11 proteins, including alpha-1-antichymotrypsin (SERPINA3), beta-2-glycoprotein 1 (APOH), ceruloplasmin (CP), complement components C5 and C9, hemoglobin subunit alpha 1 (HBA1), leucine-rich alpha-2-glycoprotein (LRG1), vitamin K-dependent protein S (PROS1), and zinc-alpha-2-glycoprotein (AZGP1). Eight multifactorial proteins—attractin (ATRN), CP, coagulation factor XII (F12), apolipoprotein A-IV (APOA4), CA1, HBA1, kininogen-1 (KNG1), and AZGP1—demonstrated dependence on two or more clinical factors.

Following MoM value adjustment (Supplementary Figure S6), the median range (5th–95th percentile) was 0.64 MoM (Supplementary Table S6), with LPA and PZP remaining the most variable proteins.

### 2.5. Proteomic Data Comparison and Validation

**Figure 3:**
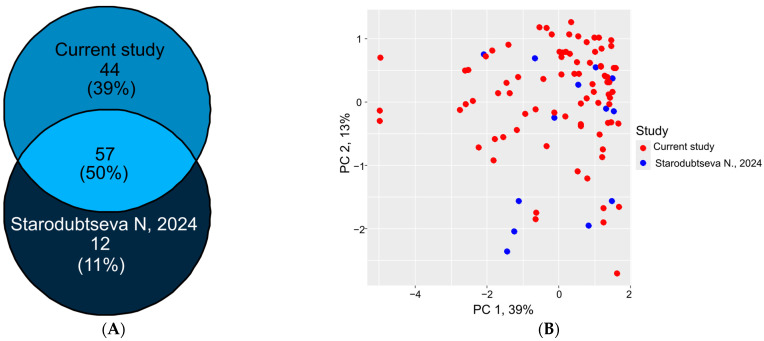
Concordance between first-trimester serum proteomes (LC-MRM-MS). (**A**) Overlap of quantified proteins between the current study (*n* = 83) and healthy pregnancies from Starodubtseva et al. (*n* = 13) [18]. (**B**) Principal component analysis of batch-adjusted and MoM-normalized proteomic profiles from both cohorts.

Our proteomic profiling (*n* = 83) demonstrated significant overlap with a previous study of first-trimester healthy pregnancies (*n* = 13) using identical LC-MRM-MS methodology with the BAK-125 kit [18]. Of the detected proteins, 57 showed consistent expression patterns between both cohorts (Figure 3A), while only 12 proteins (adipocyte plasma membrane-associated protein, alpha-1-antitrypsin, apolipoprotein A-1, apolipoprotein C-4, clusterin, complement C1q chain B, fetuin-B, inter-alpha-trypsin inhibitor heavy chain H2, tissue inhibitor of metalloproteinases 2, protein C inhibitor, tenascin-X, and thrombospondin 1) were unique to the Starodubtseva et al. study [18]. Following batch adjustment, MoM normalization, and clinical value correction, the 57 common proteins showed excellent concordance in principal component analysis (Figure 3B) and MoM-transformed values (Supplementary Figure S7).

The comparison between measurement methods revealed important insights. Routine ELISA and LC-MRM-MS values showed strong correlation (R = 0.65, *p* < 0.001) in initial analyses. This association decreased following MoM transformation (R = 0.56, *p* < 0.001) but showed slight improvement with corrected LC-MS MoM values (R = 0.58, *p*< 0.001) (Table 3).

## 3. Discussion

This prospective study characterized the serum proteome of 83 women with physiological pregnancies undergoing first-trimester screening between 11^+2^ and 13^+6^ weeks of gestation. The cohort demonstrated typical maternal characteristics, with a median age of 30.5 years and a median BMI of 21.2 kg/m^2^. The prevalences of uterine myoma (10%) and anemia (33%) were consistent with expected population rates. Fetal sex distribution was balanced (49% male), and nearly half of the women were primiparous (48%).

First-trimester biomarker profiles aligned with established norms, including MoM values of 0.95 for PAPP-A, 0.84 for PlGF, and 0.99 for free β-hCG. Hemodynamic assessments revealed normal UtA-PI (0.99 MoM) and MAP (0.98 MoM). The cohort demonstrated low baseline risks for adverse outcomes, with calculated probabilities of 1:1357 for PE, 1:554 for IUGR, and 1:2115 for preterm delivery.

Using liquid chromatography–multiple reaction monitoring–mass spectrometry (LC-MRM-MS) with stable isotope-labeled standard peptides, we successfully quantified 115 out of 139 targeted proteins (83%), demonstrating robust analytical performance. Among these, 101 proteins met all quality control criteria in accordance with ICH M10 guidelines for bioanalytical method validation and were detected in more than 50% of samples. The measured protein concentrations exhibited a wide dynamic range spanning 5.3 orders of magnitude.

Analysis of first-trimester maternal serum proteins revealed statistically significant associations with several maternal characteristics, including BMI, age, parity, fetal sex, and gestational age. Furthermore, we identified 12 proteins that demonstrated statistically significant correlations with trisomy risk, with absolute correlation coefficients ranging from 0.21 to 0.45 (*p* < 0.05). These findings highlight the potential utility of first-trimester serum protein profiling for understanding pregnancy physiology and assessing chromosomal abnormality risk.

We applied MoM transformation to serum protein concentrations to normalize for gestational age effects, account for inter-individual biological variation, and enable direct clinical interpretation through established risk assessment frameworks. This approach is well established in prenatal screening, particularly for assessing risks of Down syndrome and neural tube defects through maternal serum markers (e.g., hCG MoM values) [39,40,41]. MoM normalization facilitates cross-laboratory result comparison by accounting for methodological variations and biological differences [42].

RobNorm represents an innovative normalization approach that employs a Gaussian mixture model to characterize protein expression distributions and account for sample effects [43]. A comprehensive evaluation by Arend et al. across six spike-in and three label-free/tandem mass tag datasets demonstrated that RobNorm-normalized data exhibited intermediate performance in pooled median absolute deviation—surpassing cyclic-LOESS while approximating quantile normalization [44]. Notably, RobNorm achieved superior discriminative power for first-trimester serum proteome data compared to alternative normalization methods.

ComBat, an established batch-effect correction technique widely applied in proteomics [45], typically outperforms both quantile normalization and cyclic-LOESS in correction efficacy [46,47]. However, our current analysis revealed reduced ComBat performance, potentially attributable to size-imbalanced batch composition. Importantly, we observed comparable magnitudes of batch-induced and intra-batch variation, suggesting suboptimal algorithm performance under these specific experimental conditions—a finding that contrasts with previous successful implementations reported in the literature.

Consistent with prior research, we observed that LPA concentrations vary drastically (10-fold between pregnant women), primarily due to genetic rather than clinical influences [48]. As the most genetically controlled lipoprotein, LPA’s variability stems from its size polymorphism [49,50]. Strong evidence links high LPA to increased cardiovascular disease (CVD) risk, with recent UK Biobank data (460,000+ participants) showing a linear risk rise above median levels (19.6 nM) [51]. LPA’s structural similarity to plasminogen suggests it bridges cholesterol transport and fibrinolysis, potentially modulating clotting balance [24]. It also carries oxidized phospholipids, activating pro-inflammatory pathways in arteries [52]. Monocytes from high-LPA individuals show enhanced endothelial transmigration and cytokine production, implicating LPA in endothelial dysfunction—key in conditions like PE [53].

PZP emerged as the most variable protein in our study (fold change = 123), showing a strong association with gestational age. Our measurements revealed a PZP concentration of 388 nM (range: 7.74 nM–2.5 µM), which appears lower than previously reported values. This finding contrasts with earlier studies using different methodologies: Ekelund et al. (1994) reported mean concentrations increasing from ≈1.8 µM in the first trimester to ≈4.3 µM later in pregnancy using electroimmunoassay [54], while another study using ELISA measured ≈2.5 µM (range: 89 nM–8.9 µM) at 17–20 weeks’ gestation [55].

The extreme variability of PZP is well documented, with Ekelund’s study reporting a coefficient of variation exceeding 70% [54]. This wide dynamic range has been further corroborated by large-scale proteomic analyses, including a DIA-MS study of 253 proteins across 1800 cancer patient plasma samples, which identified both PZP and LPA as having exceptionally broad interquartile ranges [25].

Our analysis revealed CA1 as a highly dynamic protein in pregnancy, demonstrating both significant elevation (7.3-fold change) and considerable variability (CV = 70%). This pattern stands in sharp contrast to the tightly regulated CA1 levels observed in non-pregnant healthy individuals, where Gao et al. reported remarkably consistent concentrations (12.8 ± 0.7 nM by ELISA; *n* = 40) [56].

Intriguingly, our measured CA1 levels (20.9 ± 14.6 nM) more closely resembled the elevated and variable concentrations found in breast cancer patients (13.05 ± 16.25 nM by LC-MRM-MS; *n* = 183) than those in healthy controls (5.21 ± 1.97 nM; *n* = 51) from the Kim et al. study [57]. This similarity in protein dynamics between pregnancy and a pathological state, as measured by the same LC-MRM-MS methodology, suggests that CA1 may undergo similar regulation patterns in these distinct biological conditions. The substantial divergence from typical healthy control values (5.21–12.8 nM) strongly indicates that the observed CA1 profile represents a unique pregnancy-associated pattern, potentially reflecting the profound physiological adaptations occurring during gestation.

HPX demonstrated remarkable stability in our first-trimester serum proteome analysis, showing a minimal fold change of 1.3 and a remarkably low coefficient of variation (CV = 8.5%). We observed a mean HPX concentration of 13.2 µM, ranging from 10.5 to 15.9 µM. This measurement appears consistently lower than values reported in other pregnancy studies using ELISA quantification [58,59,60].

Previous studies have reported higher HPX concentrations at different gestational stages [58,59,60]. For instance, HPX levels near delivery were measured at 4.5 ± 2.2 µM in a cohort of 49 healthy women [58]. First-trimester measurements showed even higher values, with a median of ≈17.5 µM among 100 controls [59] and a mean of ≈20.04 (95% CI: 19.49–20.61) µM in a larger study of 347 healthy pregnant women at 6–20 weeks’ gestation [60].

The observed differences between our results and previous findings may be attributed to several factors. Physiological changes in HPX concentration throughout pregnancy could explain some variation, while methodological differences between mass spectrometry and immunoassays may also contribute. Additionally, population-specific variations in protein levels cannot be ruled out. Despite these quantitative differences, the exceptional stability of HPX in our dataset underscores its potential value as a reference protein for first-trimester proteomic studies.

PAPP-A serves as a clinically significant marker for various pregnancy conditions, including Down syndrome screening, intrauterine growth restriction, preeclampsia, preterm birth, and pregnancy loss [61,62,63,64]. The protein’s serum concentration demonstrates complex regulation, showing a near-exponential increase with gestational age [31] while being influenced by multiple factors, including maternal medical history, race, IVF conception, and anthropometric measurements [23,65].

In our study, parity emerged as the strongest predictor of PAPP-A levels (β = −0.17, *p* = 0.04), surpassing other potential associations. We observed a median PAPP-A concentration of ≈7.6 nM by ELISA, which closely aligned with our LC-MRM-MS quantification (6.6 nM) but was lower than the ≈10.9 nM reported by R. Caliscan et al. [66]. The methodological consistency between ELISA and mass spectrometry measurements, despite absolute concentration differences, supports the reliability of PAPP-A quantification across platforms. The observed variations highlight the need for standardized correction approaches when comparing proteomic data across different study designs and measurement techniques.

Our study presents a comprehensive quantitative analysis of first-trimester serum proteins in pregnancy using highly sensitive LC-MRM-MS with SIS peptides. From an initial screening cohort of over 1800 women, we established a well-characterized reference group of 83 healthy pregnancies through stringent exclusion criteria. The analytical platform demonstrated robust performance, reliably quantifying 115 of 139 targeted proteins (83%), with 101 proteins meeting stringent ICH M10 validation criteria and detection in >50% of samples. The quantified proteins included clinically relevant biomarkers spanning 38 FDA-approved targets, 19 CVD markers, and 25 CLIA-approved analytes, along with 23 potential PE markers. The assay’s exceptional dynamic range (5.3 orders of magnitude) enabled precise measurement across diverse protein abundance levels. The establishment of pregnancy-specific reference ranges using MoM adjustment represents a significant advance, properly accounting for gestational age and maternal characteristics that are often overlooked in conventional clinical interpretation.

Comparative analysis with our recent study, utilizing the same methodology (LC-MRM-MS with SIS) [18], validates the robustness of our proteomic approach and demonstrates high reproducibility across independent pregnancy cohorts. The strong concordance in protein expression patterns (50% overlap) despite different sample sizes supports the reliability of our findings. These results establish a foundation for future large-scale validation of pregnancy protein biomarkers, with particular emphasis on the 57 consistently expressed proteins as potential core markers for first-trimester screening applications. The successful normalization across studies further confirms the utility of MoM transformation for cross-study proteomic comparisons in pregnancy research.

Several important limitations should be considered when interpreting these findings. The sample size of 83 healthy pregnancies, while carefully selected from a larger screening population, may constrain the generalizability of results, particularly for rare pregnancy complications. The observed discrepancies between LC-MRM-MS and ELISA measurements highlight important methodological considerations—while mass spectrometry provides superior specificity, absolute concentration differences may reflect both technical variations between platforms and inherent cohort characteristics.

The moderate attenuation in correlation between LC-MS and ELISA measurements following MoM transformation (R = 0.58) for PAPP-A indicates that while this normalization approach enhances cross-platform comparability, further optimization may be necessary to achieve complete analytical harmonization. Notably, the 12 candidate protein markers for trisomy risk identified in this study will require extensive validation in large-scale prospective studies before consideration for clinical application.

Despite these limitations, our validated proteomic framework establishes reference data for 101 first-trimester serum biomarkers, including 38 with existing FDA-cleared ELISA kits for immediate clinical integration. For broader implementation, multiplex platforms (e.g., Luminex/MSD) could enable efficient screening of comprehensive protein panels in prenatal care.

## 4. Materials and Methods

### 4.1. Study Design

This prospective cohort study was conducted at the I. Kulakov National Medical Research Center for Obstetrics, Gynecology, and Perinatology between January 2022 and December 2022. From an initial screening population of 1869 women aged 18–45 years undergoing routine first-trimester assessment (11^+2^ to 13^+6^ weeks’ gestation), we selected a final analytical cohort of 83 women (4.4% of screened participants) who met stringent criteria for uncomplicated singleton pregnancies. These controls were specifically chosen to establish reference values for normal pregnancy progression.

The selection process involved multiple stages of rigorous screening. We restricted our analysis to singleton pregnancies to maintain cohort homogeneity and avoid confounding effects from multifetal gestations, which exhibit systematically different serum protein profiles due to greater hemodilution and altered placental biomarker production [67,68,69]. This approach aligns with established proteomic research standards by eliminating known physiological differences that could bias protein concentration analyses [70]. Participants were excluded if their pregnancy resulted from assisted reproductive technology or if they had any of the following conditions: pre-existing diabetes mellitus, autoimmune disorders, organ transplantation history, or malignancies. We further excluded cases that subsequently developed pregnancy complications, including fetal chromosomal abnormalities, PE, gestational hypertension (≥140/90 mmHg on two measurements), gestational diabetes mellitus (diagnosed by oral glucose tolerance testing), preterm delivery (<37 + 0 weeks), or IUGR (estimated fetal weight <10th percentile).

All participants provided written informed consent prior to enrollment. The first-trimester screening protocol included comprehensive maternal evaluation with standardized measurements of weight, height, and blood pressure [71]. Transabdominal color Doppler ultrasound was performed to measure the UtA-PI [72]. Serum concentrations of PIGF and PAPP-A were quantified using the Delfia Xpress system (PerkinElmer Life and Analytical Sciences, Shelton, CT, USA) according to manufacturer protocols. The final control group comprised women who maintained uncomplicated pregnancies through term delivery (≥37 weeks) with appropriate fetal growth (birthweight 10th–90th percentiles).

The study protocol was approved by the Institutional Review Board of the National Medical Research Center for Obstetrics, Gynecology, and Perinatology (protocol No. 2, dated 9 March 2017). All procedures were conducted in accordance with the ethical standards of the Helsinki Declaration and Good Clinical Practice guidelines.

### 4.2. Sample Preparation

We analyzed serum samples from 83 singleton pregnancies collected during routine first-trimester screening (11^+2^ to 13^+6^ weeks’ gestation). Blood was drawn into SerumZ/9 tubes (Monovette, Sarstedt, Germany) and processed within 2 h of collection. After centrifugation (300× *g*, 20 min, room temperature), the supernatant was aliquoted into 200 μL portions in cryo-tubes and stored at −80 °C until analysis.

The study employed 139 SIS (“heavy”) peptides as internal standards and 139 corresponding unlabeled NAT (“light”) peptides for calibration. Synthesis and characterization of SIS and NAT peptides was carried out in the Omics lab at Skoltech using standard procedures, which were previously described in detail [8,73]. The assay was adapted from the BAK-270 kit (MRM Proteomics Inc., Montreal, QC, Canada) [8,73,74,75].

The SIS peptide mixture was added uniformly to all samples (10 μL per sample at 100 fmol/μL final concentration), including the 83 clinical samples, 7-level calibration standards (A-G), and 4 quality controls (QCA-C and CLP). In contrast, the NAT peptides were exclusively added to the calibration standards at seven different concentrations spanning from LLOQ (level A) to HLOQ (level G). Sample preparation followed established protocols [8,20,73], starting with 10 μL of serum. Proteins were denatured and reduced using 9 M urea, 20 mM dithiothreitol, and 200 mM Tris × HCl (pH 8.0, 37 °C, 30 min), then alkylated with 100 mM iodoacetamide (30 min, dark). For trypsinolysis, samples were diluted with 100 mM Tris × HCl (pH 8.0) to <1 M urea, treated with TPCK-trypsin (20:1 *w*/*w*), and incubated (18 h, 37 °C). The reaction was quenched with formic acid (1.0% final, pH ≤ 2), yielding ~1 mg/mL peptides. After SIS peptide addition, samples underwent solid-phase extraction (SPE) cleanup and were reconstituted in 34 μL of 0.1% formic acid (FA) for LC-MRM-MS.

Two distinct types of quality controls were implemented throughout the study. The BSA-based QCs (QCA-C) represented three concentration levels (low, medium, and high) prepared in a surrogate matrix, while the pooled serum QC (CPL) served to monitor batch effects. Process blanks were included to assess potential background interference. All QCs and calibration standards underwent identical processing as the clinical samples.

### 4.3. LC-MRM-MS Analysis

The LC-MRM-MS analysis was performed across three batches using an ExionLC UHPLC system (ThermoFisher Scientific, Waltham, MA, USA), coupled to a SCIEX QTRAP 6500+ mass spectrometer (SCIEX, Toronto, ON, Canada). Samples (*n* = 83) were analyzed in randomized duplicate, with each 96-well plate containing a complete set of calibration standards (A-G), QCs (QCA-C and CLP), and blanks.

Chromatographic separation used a C18 column (2.1 × 150 mm, 1.7 μm) with a 53 min 2–45% acetonitrile gradient (0.4 mL/min, 0.1% FA) [73]. Mass spectrometric detection was performed in positive electrospray ionization mode with the ion source operated at 4000 V and 450 °C, monitoring the optimized transitions detailed in Supplementary Table S8.

The analytical batches were designed to ensure data quality, with calibration standards analyzed at the start of each batch, followed by QCs distributed throughout the run (beginning, middle, and end) to monitor system performance.

### 4.4. Data Preprocessing, Quality Control, and Quantitative Analysis

Raw MS data preprocessing, including quantitative analysis, was performed in Skyline software (version 20.2.0.343, University of Washington) [76]. Data quality evaluation and quantitation were conducted in compliance with the ICH guidelines for Bioanalytical Method Validation [34]. Calibration standards, consisting of seven levels (A–G), along with QC (QCA-C and CPL) samples, were employed to evaluate assay performance. Calibration curves were generated using 1/(x × x)-weighted linear regression methods to calculate the peptide and corresponding protein concentrations in the measured samples (fmol per 1 µL of plasma).

The acceptance criteria required that both accuracy and precision remain within 20% of the theoretical value, with calculated concentrations deviating no more than ±20% from their expected values. A calibration curve was deemed acceptable provided that a minimum of five out of the eight standards met these criteria. Furthermore, for the experiment to be considered valid, at least 66% of all QC samples and no less than 90% of the peptide calibration curves were required to fulfill the acceptance criteria. Proteins demonstrating calibration curves with R^2^ > 0.99 were classified as quantified. For sample measurements falling below the lower limit of quantification (LLOQ, level A), the LLOQ value was assigned. Similarly, measurements exceeding the upper limit of quantification (HLOQ, level G) were assigned the HLOQ value. Additional exclusion criteria comprised proteins with concentrations below LLOQ or above HLOQ in more than 50% of study samples.

### 4.5. Statistical Analysis and Normalization

To minimize batch effects and analytical variance, four normalization approaches were evaluated and compared: quantile normalization [77], LOESS normalization [78,79], Robnorm [43], and ComBat normalization [80]. For each normalized dataset along with the raw data, CVs were calculated across standard and quality control samples. Proteins exhibiting a mean CV ≥ 20% were subsequently excluded from further analysis.

The processed datasets were autoscaled and visualized through principal component analysis. Statistical characterization was performed for both raw and optimally normalized data, including determination of median values, standard deviations, coefficients of variation, minimum/maximum values, and 5%/95% quantile levels. The fold change was computed as the ratio between the 95% and 5% quantile values.

### 4.6. Reference Value Establishment and Clinical Parameter Association

The relationship between normalized protein concentrations and first-trimester screening parameters was assessed using Spearman’s rank correlation analysis, applicable to continuous and categorical variables, with statistical significance set at *p* < 0.05. For quantitative analysis, protein concentrations were expressed as MoM, calculated by dividing each measured concentration (*C^i,j^*) by the median concentration of the corresponding protein across all samples:

$$C_{MoM}^{i,j}=\frac{C^{i,j}}{median(C^{i})}$$

where *C^i,j^* is the concentration of protein *i* in the sample *j*.

Multiple linear regression modeling was employed to examine associations between protein MoM values and clinical parameters, including maternal age, body mass index, gestational age at blood collection, parity, and uterine myoma status. Each protein was analyzed through an iterative model refinement process, where non-significant predictors (*p* ≥ 0.05) were sequentially removed starting with the least significant variable.

For each final model, we determined the clinical parameter values corresponding to the baseline protein expression level (1 MoM). These models were subsequently used to derive adjustment factors for protein concentration normalization.

All statistical computations and data transformations were performed using custom R scripts (version 4.3.3) incorporating the following packages: Sva (v3.50.0) for surrogate variable analysis [81], limma (v3.58.1) for linear modeling [82], reshape2 (v1.4.4) for data manipulation [83], ggplot2 (v3.5.2) for visualization [84], and pheatmap (v1.0.12) for heatmap generation [85].

## 5. Conclusions

This prospective study provides a comprehensive characterization of the first-trimester serum proteome in 83 women with singleton physiological pregnancies, establishing robust reference ranges for 101 rigorously validated proteins. Using our highly sensitive LC-MRM-MS platform, we achieved reliable protein quantification across an impressive 5.3-order-of-magnitude dynamic range, revealing significant associations between protein expression patterns and key maternal–fetal characteristics, including BMI, age, parity, and fetal sex, as well as potential markers of trisomy risk. Through careful evaluation of normalization methods, we determined that the RobNorm approach most effectively addressed inter-batch variation in our dataset.

Among the study’s most significant findings are the identification of twelve candidate protein markers for chromosomal abnormalities, the successful application of MoM transformation for cross-platform data harmonization, and the discovery of striking variability in pregnancy-associated proteins such as PZP, LPA, PAPP-A, and CA1. These proteins demonstrate patterns that may reflect their unique roles in gestational physiology and pathology. Particularly noteworthy is CA1’s dynamic behavior, which closely resembles patterns observed in malignant conditions, while HPX exhibited remarkable stability, suggesting its potential utility as a reference protein in pregnancy studies.

The study also revealed important methodological considerations. While MoM transformation improved comparability between platforms, the moderate post-transformation correlation (R = 0.58) for PAPP-A measurements highlights ongoing challenges in cross-method standardization. This finding emphasizes the need for continued refinement of normalization strategies to enable more robust integration of proteomic data across different analytical platforms.

Although the study’s moderate sample size and observed methodological variability represent limitations that warrant further investigation in larger cohorts, the current findings make substantial contributions to the field. The establishment of pregnancy-specific reference ranges for fifty-seven consistently detected proteins, including numerous FDA- and CLIA-approved biomarkers, creates a valuable resource for future research. The validated LC-MRM-MS workflow, which meets stringent ICH M10 standards, along with the identification of promising candidate biomarkers, provides a strong foundation for advancing prenatal screening.

Looking ahead, priority should be given to large-scale validation of the identified protein markers in diverse populations, deeper investigation of their biological mechanisms, and development of optimized analytical pipelines. These efforts will be crucial for translating these discoveries into clinical applications. By significantly expanding our understanding of first-trimester proteomic dynamics and demonstrating practical approaches to data standardization, this work represents an important step toward more precise and comprehensive prenatal care.

## Figures and Tables

**Table 1:** Clinical characteristics of women with physiological pregnancies undergoing first-trimester screening (*n* = 83). Continuous variables are reported as median (1st quartile; 3rd quartile) with minimum and maximum values. Categorical variables are presented as frequency counts and percentages within the group. BMI—body mass index, PAPP-A—pregnancy-associated plasma protein A, PlGF—placental growth factor, hCG—human chorionic gonadotropin, MoM—multiples of the median, MAP—mean arterial pressure, UtA-PI—pulsatility index of the left and right uterine arteries, PE—preeclampsia, IUGR—intrauterine growth restriction.

Clinical Characteristic	Value
Age, years	30.5 (27.4; 32.8) 20.5–37.3
BMI, kg/m^2^	21.2 (19.2; 23.0) 15.6–30.1
Gestational age at blood collection, weeks	12.4 (12.1; 12.9) 11.3–13.9
Gestational age at delivery, weeks	39.5 (39; 40.2) 37.5–41.2
Uterine myoma, *n* (%)	8 (10%)
Anemia during pregnancy, *n* (%)	27 (33%)
Fetal sex (male)	41 (49%)
Parity, *n* (%)	1–40 (48%) 2–32 (39%) 3–10 (12%) 4–1 (1%)
1st screening	
PAPP-A, mLU/mL	3.03 (2.08; 4.59) 0.598–9.761
PAPP-A, MoM	0.95 (0.65; 1.47) 0.196–4.271
PlGF, pg/ml	25.3 (20.2; 35) 13.3–54
PlGF, MoM	0.84 (0.61; 1.03) 0.374–1.521
free β-HGC, ng/ml	52.61 (37.38; 75.54) 12.57–224.31
free β-HGC, MoM	0.99 (0.78; 1.2) 0.533–1.667
UtA-PI	1.57 (1.29; 2.02) 0.89–2.655
UtA-PI, MoM	0.99 (0.78; 1.2) 0.533–1.667
MAP, mmHg	83.33 (77.46; 86.46) 66–98.833
MAP, MoM	0.98 (0.94; 1.04) 0.8027–1.1755
Risk of PE	1357.5 (516.75; 2816.5) 63–15,320
Risk of IUGR	554 (377; 877) 81–2501
Risk of preterm delivery	2115.5 (890; 3209.75) 5–5026
Risk of 21th trisomy (background)	618 (405; 830) 159–1108
Risk of 21th trisomy (adjusted)	9997 (5206; 15,160) 227–22,160
Risk of 18th trisomy (background)	1471 (976; 2115) 381–2787
Risk of 18th trisomy (adjusted)	2870 (16,161; 41,012) 409–55,750
Risk of 13th trisomy (background)	4589 (3065; 6609) 1197–8722
Risk of 13th trisomy (adjusted)	83,050 (45,488; 122,252) 9180–174,431

**Table 2:** List of proteins exhibiting statistically significant (*p* < 0.05) linear relationships with maternal/fetal clinical characteristics. Proteins dependent on two or more clinical factors are highlighted in bold.

Clinical	Direction of Association	Protein (Gene Name)
BMI	direct	**ATRN**, CA4BPA, **CP**, **F12**, C1QA, C1R, C3, CFB, CFI, HP, HABP2, APCS
	reverse	AHSG, SERPINC1, **APOA4**, APOD, **CA1**, **HBA1**, IGFBP3, SERPING1, **AZGP1**
Age	direct	SERPIND1
	reverse	A2M
Parity	direct	AGT, APOC3, CNDP1, **CA1**, IGHG1, **KNG1**, PLG
	reverse	**ATRN**, **F12**, SERPINA6, SELL, PAPP-A, ALB
Gestational age at blood collection	direct	VTN
	reverse	HRG
Uterine myoma	direct	**KNG1**
	reverse	**APOA4**, SPARC
Male fetal sex	direct	BTD, **CA1**
	reverse	SERPINA3, APOH, **CP**, C5, C9, **HBA1**, LRG1, PROS1, **AZGP1**

**Table 3:** Association according to Pearson’s test between PAPP-A levels, quantified and processed by two methods.

Screening	LC-MS	R	*p*-Value
3.03 (2.08; 4.59) IU/mL	6.59 (4.84; 8.82) nM	0.65	<0.001
0.95 (0.65; 1.47) MoM	6.59 (4.84; 8.82) nM	0.56	<0.001
0.95 (0.65; 1.47) MoM	0.95 (0.65; 1.28) MoM	0.58	<0.001

## Data Availability

All experimental LC-MRM-MS results were uploaded to the PeptideAtlas SRM Experiment Library (PASSEL) and are available via the following link: ftp://PASS05921:RZ8565pk@ftp.peptideatlas.org/ (publicly accessible on 1 September 2025).

## Supplementary Materials

The following supporting information can be downloaded at https://www.mdpi.com/article/10.3390/ijms26167970/s1.

## Abbreviations

The following abbreviations are used in this manuscript:

BMI	Body mass index
PE	Preeclampsia
IUGR	Intrauterine growth restriction
SIS	Stable isotope-labeled standards
NAT	Natural synthetic proteotypic peptides
CV	Coefficient of variation
DIA	Data-independent acquisition
ELISA	Enzyme-linked immunosorbent assay
PEA	Proximity extension assay
HLOQ	The highest limit of quantification
UHPLC	Ultra-high-performance liquid chromatography
LC-MS	Liquid chromatography–mass-spectrometry
LLOQ	The lowest limit of quantification
LOESS	Locally estimated scatterplot smoothing
MoM	Multiply of medians
MRM	Multiply reaction monitoring
MS	Mass spectrometry
QC	Quality control
SPE	Solid-phase extraction
MAP	Mean arterial pressure
UtA-PI	Pulsatility index of the left and right uterine arteries
