# Supplementary material for: Multiplexed Quantification of First-Trimester Serum Biomarkers in Healthy Pregnancy
Source: Int J Mol Sci. 2025 Aug 18;26(16):7970. doi: 10.3390/ijms26167970 (PMC12386716; doi:10.3390/ijms26167970)
Supplement: Supplementary file 1 [file ijms-26-07970-s001.zip › Supplementary.pdf]

Supplementary

Multiplexed Quantification of First-Trimester Serum Biomarkers in Healthy Pregnancy

Natalia Starodubtseva[1]*, Alisa Tokareva[1], Alexey Kononikhin[1,2], Alexander Brzhozovskiy[1,2], Anna Bugrova[1,3], Evgenii Kukaev[1,4,5], Alina Poluektova[1], Vladimir Frankevich[1,6], Evgeny Nikolaev[2]* and Gennady Sukhikh[1,7]

1 V.I. Kulakov National Medical Research Center for Obstetrics Gynecology and Perinatology, Ministry of Healthcare of Russian Federation, 117997 Moscow, Russia; n_starodubtseva@oparina4.ru (N.S.); alisa.tokareva@phystech.edu (A.T.); alex.kononikhin@gmail.com (A.K); a_poluektova@oparina4.ru (A.P.); agb.imbp@gmail.com (A.Br.); a_bugrova@oparina4.ru (A.B.); e_kukaev@oparina4.ru (E.K.); g_sukhikh@oparina4.ru (G.S.)

2 Project Center of Omics Technologies and Advanced Mass Spectrometry, 121205 Moscow, Russia; ennikolaev@gmail.com (E.N.)

3 Emanuel Institute of Biochemical Physics, Russian Academy of Sciences, 119334 Moscow, Russia

4 V.L. Talrose Institute for Energy Problems of Chemical Physics, N.N. Semenov Federal Research Center for Chemical Physics, Russian Academy of Sciences, 119334 Moscow, Russia

5 Moscow Center for Advanced Studies, 123592 Moscow, Russia

6 Laboratory of Translational Medicine, Siberian State Medical University, 634050 Tomsk, Russia

7 Department of Obstetrics, Gynecology, Perinatology and Reproductology, Institute of Professional Education, Federal State Autonomous Educational Institution of Higher Education I.M. Sechenov First Moscow State Medical University of the Ministry of Health of the Russian Federation, 119991 Moscow, Russia

* Correspondence: n_starodubtseva@oparina4.ru; ennikolaev@gmail.com

TABLE OF CONTENT

Figure S1. Protein variability assessment using mean CV in calibration standards (A–H) and quality controls (QCA-C and pooled sample serum, CLP) under five data processing approaches: (A) raw data, (B) quantile normalization, (C) LOESS normalization, (D) RobNorm normalization, and (E) ComBat normalization. The red dashed line represents the study's maximum permitted CV threshold (20%), highlighting proteins exceeding acceptable variability.

Figure S2. Visualization of normalization efficacy on batch effects using principal component analysis: (A) raw data exhibits strong batch clustering, while (B) LOESS and (C) RobNorm normalization show progressively improved sample mixing, indicating effective technical artifact removal.

Figure S3. Raw protein concentration distributions in the 1st serum of healthy pregnant women (n=83): (A) Boxplots display protein concentration distributions. Central horizontal lines represent medians, box boundaries indicate the 1st and 3rd quartiles (interquartile range, IQR), and whiskers (lower whisker is the higher value between minimum x and Q1 – 1.5 *IQR and higher whisker is the lower value between maximum x and Q3 + 1.5*IQR)); (B) Variability analysis (95th:5th percentile ratios; threshold=3, red line) (n=83)

Figure S4. Reference ranges for first-trimester maternal serum proteins. Quantitative analysis of 101 serum proteins was performed in 83 singleton pregnancies (11^{+2} to 13^{+6} weeks gestation) using targeted mass spectrometry. Boxplots display protein concentration distributions after multiples of the median (MoM) normalization. Central horizontal lines represent medians, box boundaries indicate the 1st and 3rd quartiles (interquartile range, IQR), and whiskers (lower whisker is the higher value between minimum x and Q1 – 1.5 *IQR and higher whisker is the lower value between maximum x and Q3 + 1.5*IQR).

Figure S5. Correlation analysis of first-trimester serum proteins with clinical pregnancy parameters. Spearman's rank correlation coefficients (R) between RobNorm-normalized serum protein levels and key clinical variables are shown for statistically significant associations ($p<0.05$). Analyzed parameters include: (1) FMF algorithm-derived risks for PE, IUGR, preterm birth, and fetal aneuploidies (trisomy 21/18/13), presented as both background (_b) and adjusted (_a) risk calculations; (2) uterine artery Doppler measurements (pulsatility index, UtA-PI); and (3) maternal hemodynamic parameters (mean arterial pressure, MAP). Correlation strength is represented by color intensity, with positive (red) and negative (blue) associations indicated.

Figure S6. Adjusted multiples of the median (MoM) values for 101 serum proteins measured in 83 singleton pregnancies (11+2 to 13+6 weeks gestation) are shown in relation to six key clinical parameters: maternal body mass index (BMI), age, parity, fetal sex, gestational age at blood collection, and presence of uterine myoma. Boxplot elements represent: center line (median), box limits (IQR;25th-75th percentiles), whiskers (lower whisker is the higher value between minimum x and Q1 – 1.5 *IQR and higher whisker is the lower value between maximum x and Q3 + 1.5*IQR).

Figure S7. Concordance analysis of first-trimester serum protein profiles across studies. Comparison of MoM-normalized values for 57 common serum proteins between the current study (n=83) and Starodubtseva et al. (n=13) after batch effect correction, MoM transformation, and adjustment for clinical covariates. Box plot elements represent: center line (median), box limits (IQR; 25th-75th percentiles), and whiskers (lower whisker is the higher value between minimum x and Q1 – 1.5 *IQR and higher whisker is the lower value between maximum x and Q3 + 1.5*IQR). Protein concentrations were quantified using targeted mass spectrometry and normalized to account for inter-study technical variability and biological differences. The high degree of distributional overlap demonstrates methodological consistency in protein quantification across platforms and populations.

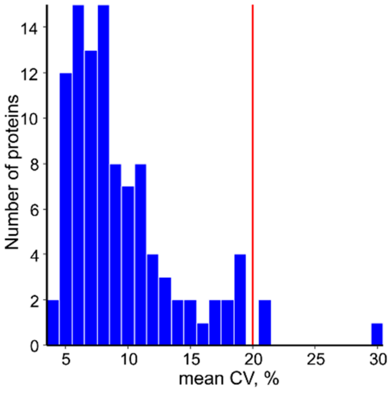

)

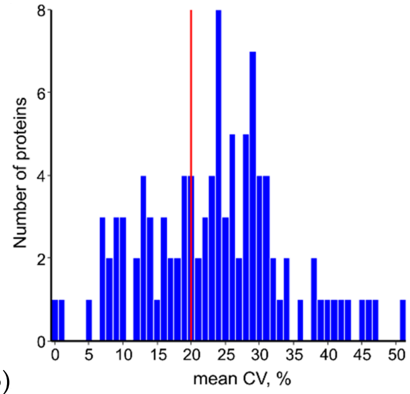

(B)

(C)

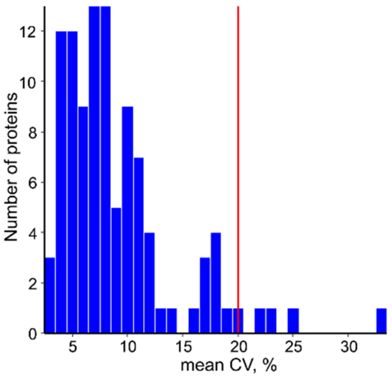

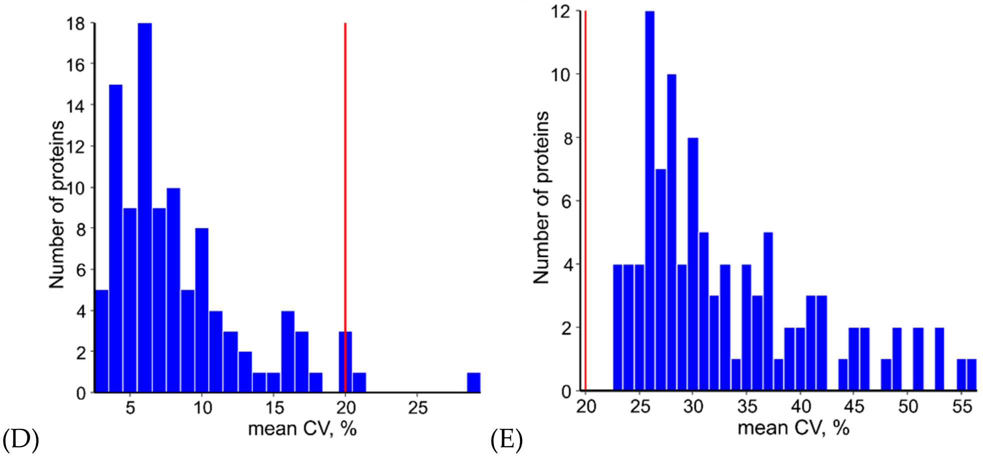

Figure S1. Protein variability assessment using mean CV in calibration standards (A–H) and quality controls (QCA-C and pooled sample serum, CLP) under five data processing approaches: (A) raw data, (B) quantile normalization, (C) LOESS normalization, (D) RobNorm normalization, and (E) ComBat normalization. The red dashed line represents the study's maximum permitted CV threshold (20%), highlighting proteins exceeding acceptable variability.

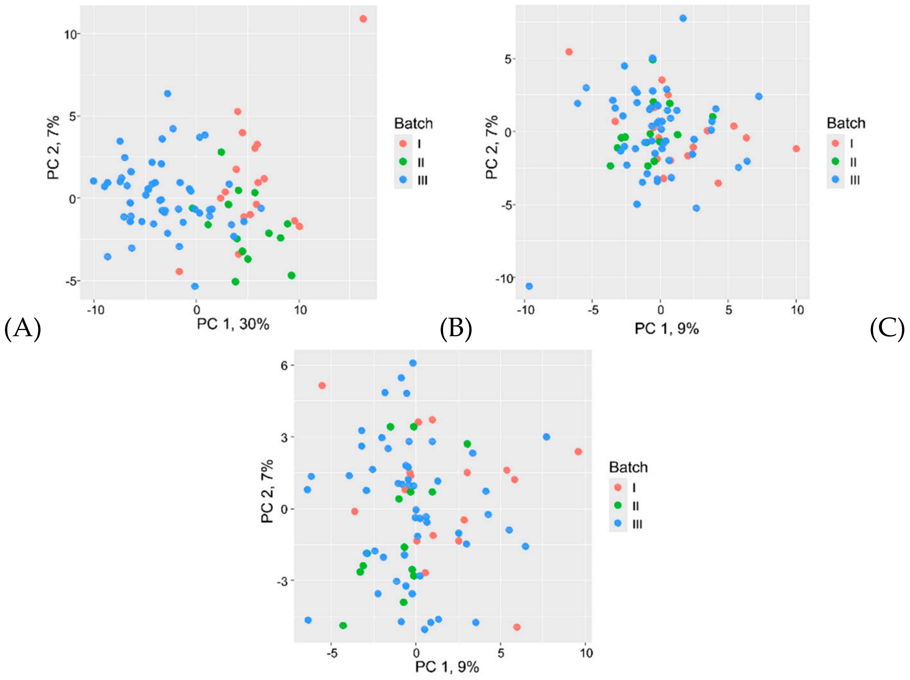

Figure S2. Visualization of normalization efficacy on batch effects using principal component analysis: (A) raw data exhibits strong batch clustering, while (B) LOESS and (C) RobNorm normalization show progressively improved sample mixing, indicating effective technical artifact removal.

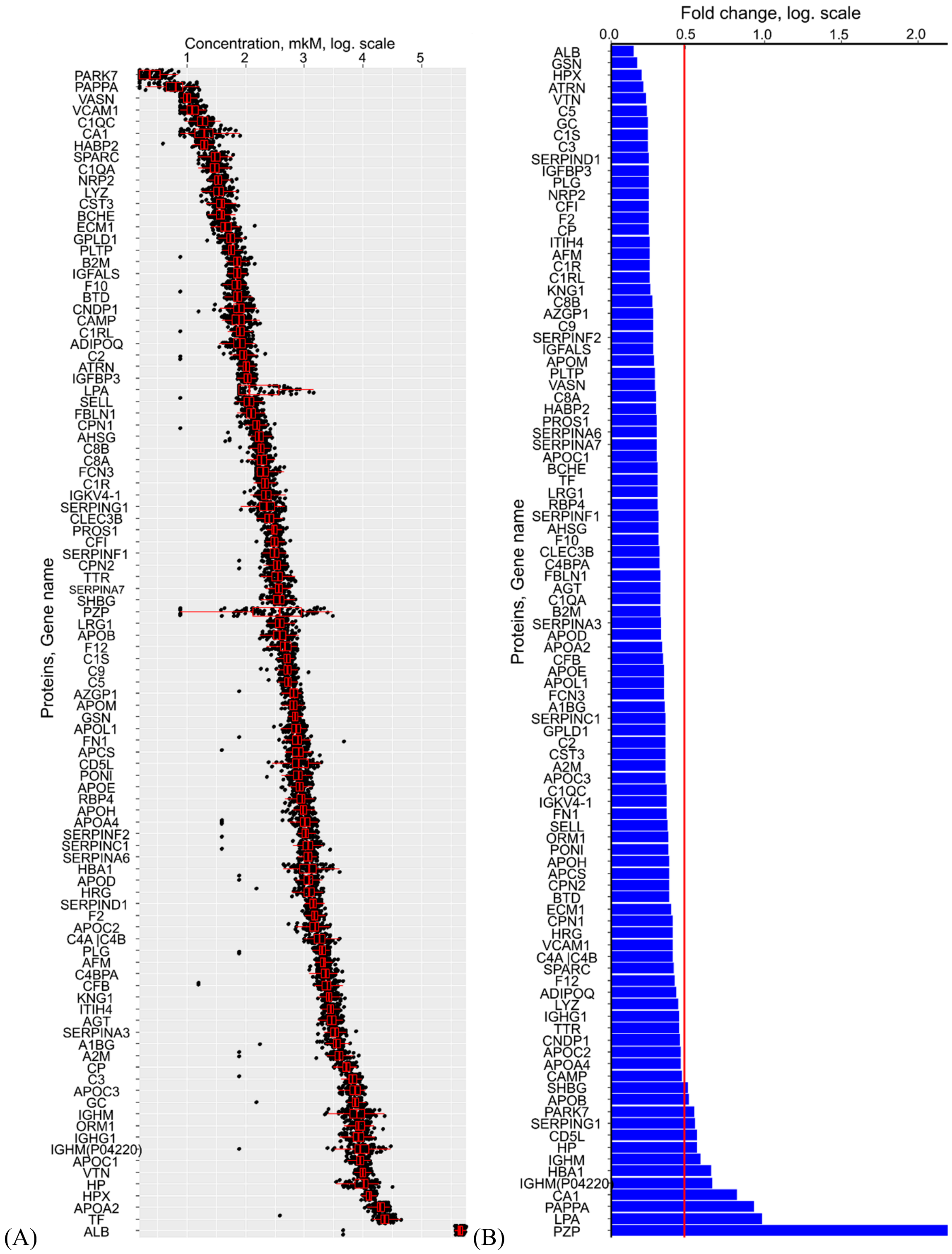

Figure S3. Raw protein concentration distributions in the 1st serum of healthy pregnant women (n=83): (A) Boxplots display protein concentration distributions. Central horizontal lines represent medians, box boundaries indicate the 1st and 3rd quartiles (interquartile range, IQR), and whiskers (lower whisker is the higher value between minimum x and Q1 – 1.5 *IQR and higher whisker is the lower value between maximum x and Q3 + 1.5*IQR)); (B) Variability analysis (95th:5th percentile ratios; threshold=3, red line) (n=83).

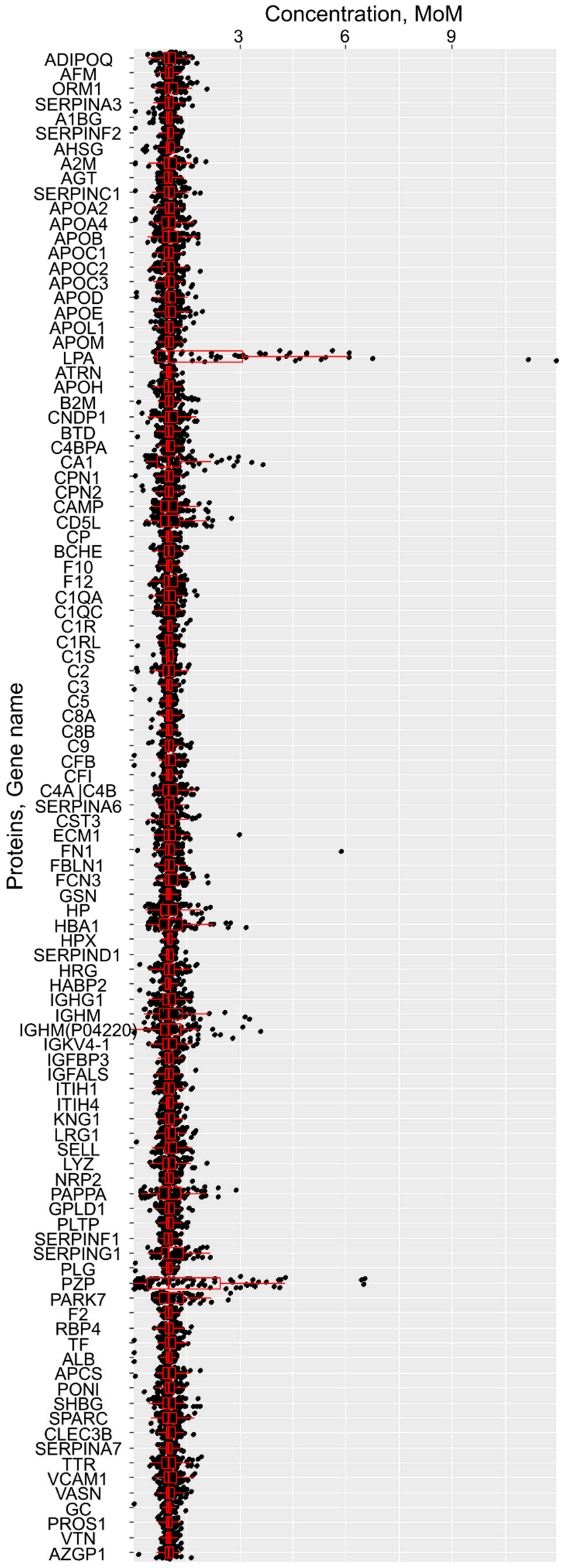

Figure S4. Reference ranges for first-trimester maternal serum proteins. Quantitative analysis of 101 serum proteins was performed in 83 singleton pregnancies (11^{+2} to 13^{+6} weeks gestation) using targeted mass spectrometry. Box plots display protein concentration distributions after multiples

of the median (MoM) normalization. Central horizontal lines represent medians, box boundaries indicate the 1st and 3rd quartiles (interquartile range, IQR), and whiskers (lower whisker is the higher value between minimum x and Q1 – 1.5 *IQR and higher whisker is the lower value between maximum x and Q3 + 1.5*IQR).

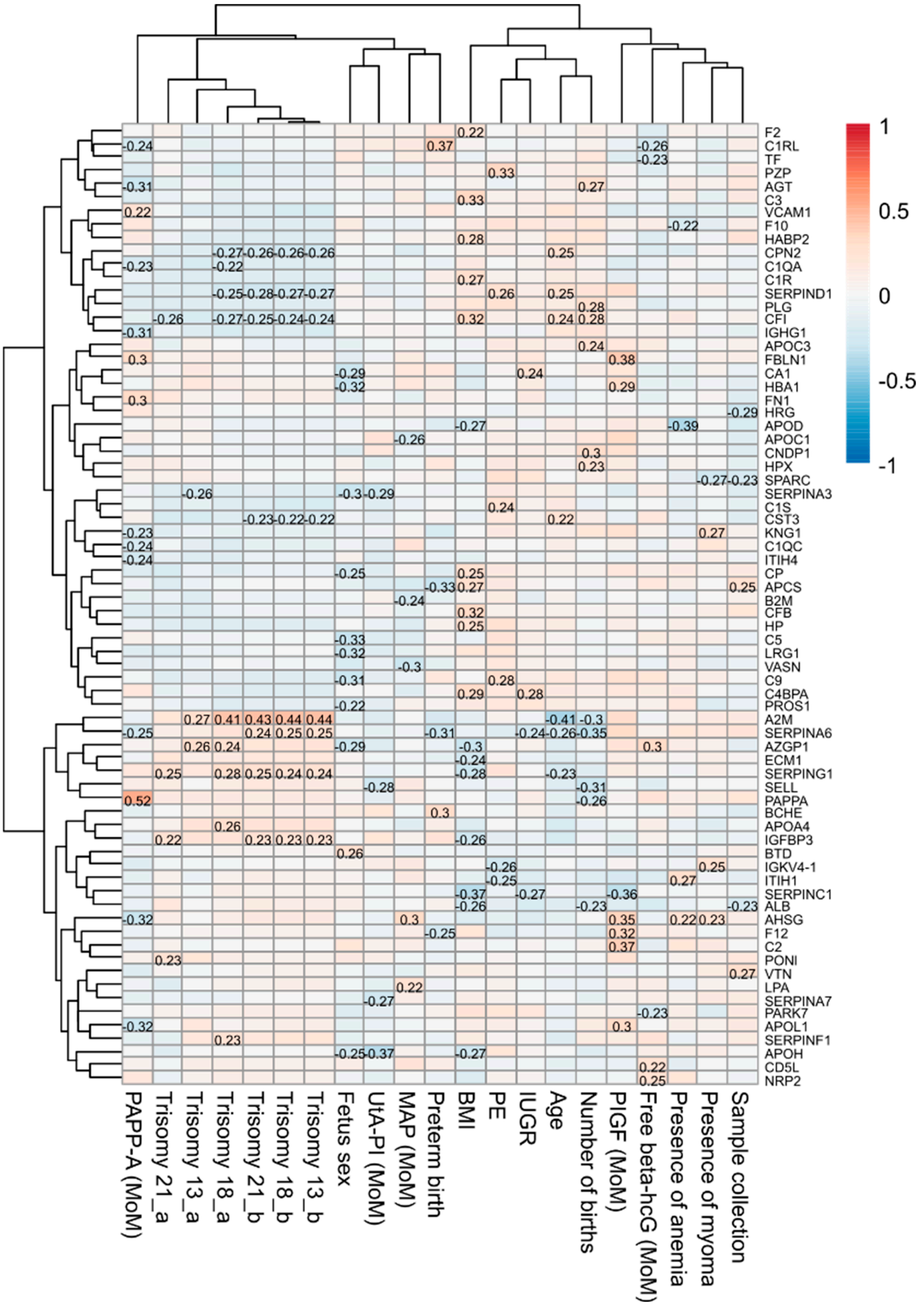

Figure S5. Correlation analysis of first-trimester serum proteins with clinical pregnancy parameters. Spearman's rank correlation coefficients (R) between RobNorm-normalized serum protein levels and key clinical variables are shown for statistically significant associations (p<0.05). Analyzed parameters include: (1) FMF algorithm-derived risks for PE, IUGR, preterm birth, and fetal aneuploidies (trisomy 21/18/13), presented as both background (_b) and adjusted

(_a) risk calculations; (2) uterine artery Doppler measurements (pulsatility index, UtA-PI); and (3) maternal hemodynamic parameters (mean arterial pressure, MAP). Correlation strength is represented by color intensity, with positive (red) and negative (blue) associations indicated.

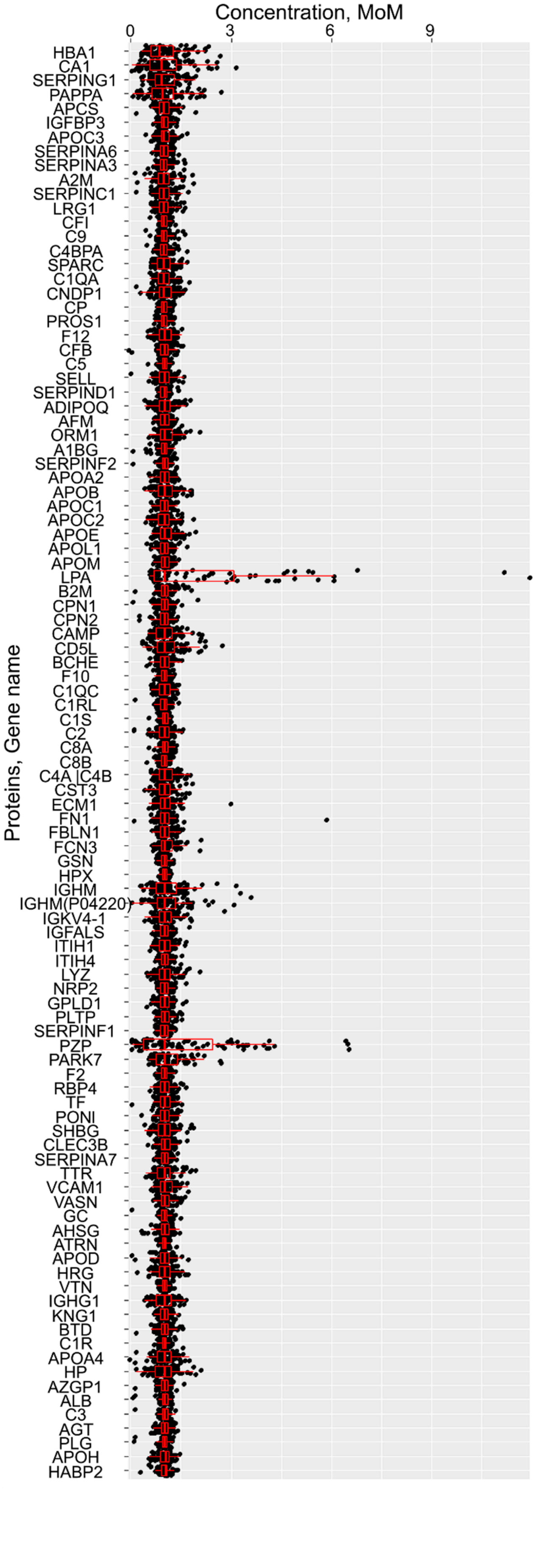

Figure S6. Adjusted multiples of the median (MoM) values for 101 serum proteins measured in 83 singleton pregnancies (11^{+2} to 13^{+6} weeks gestation) are shown in relation to six key clinical parameters: maternal body mass index (BMI), age, parity, fetal sex, gestational age at blood collection, and presence of uterine myoma. Box plot elements represent: center line (median), box limits (IQR;25th-75th percentiles), whiskers (lower whisker is the higher value between minimum x and Q1 – 1.5 *IQR and higher whisker is the lower value between maximum x and Q3 + 1.5*IQR).

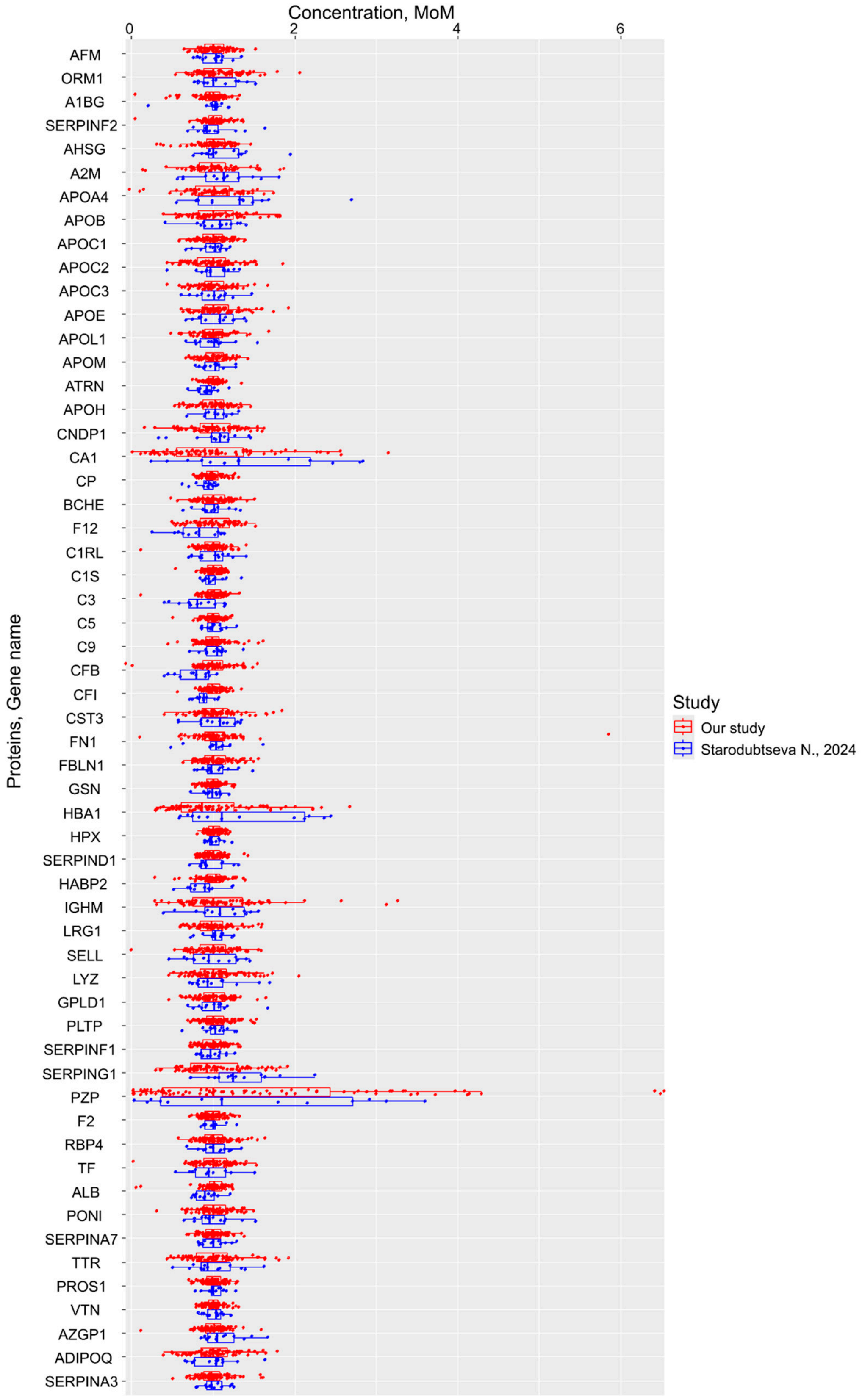

Figure S7. Concordance analysis of first-trimester serum protein profiles across studies. Comparison of MoM-normalized values for 57 common serum proteins between the current study (n=83) and Starodubtseva et al. (n=13) [1] after batch effect correction, MoM transformation, and

adjustment for clinical covariates. Box plot elements represent: center line (median), box limits (IQR; 25th-75th percentiles), and whiskers (lower whisker is the higher value between minimum x and Q1 – 1.5 *IQR and higher whisker is the lower value between maximum x and Q3 + 1.5*IQR). Protein concentrations were quantified using targeted mass spectrometry and normalized to account for inter-study technical variability and biological differences. The high degree of distributional overlap demonstrates methodological consistency in protein quantification across platforms and populations.

References

1. Starodubtseva, N.; Tokareva, A.; Kononikhin, A.; Brzhozovskiy, A.; Bugrova, A.; Kukaev, E.; Muminova, K.; Nakhabina, A.; Frankevich, V.E.; Nikolaev, E.; et al. First-Trimester Preeclampsia-Induced Disturbance in Maternal Blood Serum Proteome: A Pilot Study. *Int. J. Mol. Sci.* **2024**, *25*, 10653, doi:10.3390/ijms251910653.
